# Reliability and validity of clinically useful depression outcome scale identifying mixed features in patients with manic episode

**DOI:** 10.1002/brb3.2313

**Published:** 2021-08-01

**Authors:** Xujuan Li, Yue Fei, Haichen Yang, Wenfei Li, Zhenghui Yi, Bixiu Yang, Leping Huang, Yu Wang, Binxun Jiang, Zuowei Wang

**Affiliations:** ^1^ Shulan (Hangzhou) Hospital Affiliated to Zhejiang Shuren University Shulan International Medical College Hangzhou P. R. China; ^2^ Hongkou District Mental Health Center Shanghai P. R. China; ^3^ Shenzhen Mental Health Centre Shenzhen P. R. China; ^4^ Anhui Mental Health Center Hefei P. R. China; ^5^ Shanghai Mental Health Center Shanghai Jiao Tong University School of Medicine Shanghai P. R. China; ^6^ Wuxi Mental Health Center Wuxi P. R. China; ^7^ School of Medicine Shanghai University Shanghai P. R. China

**Keywords:** CUDOS, cut‐off score, manic episode, mixed features, psychometrics, reliability and validity

## Abstract

**Objectives:**

This study aims to explore the reliability, validity, and feasibility of Clinically Useful Depression Outcome Scale (CUDOS) in screening mixed features in patients diagnosed with mania.

**Methods:**

A total of 109 patients with (hypo‐) manic episode were recruited. The reliability of Chinese version of CUDOS (CUDOS‐C) were analyzed with Cronbach's alpha and intraclass correlation coefficient (ICC). Spearman correlation coefficient was used to analyze the validity by comparing the correlation between CUDOS‐C and Patient Health Questionnaire‐9 (PHQ‐9), 32‐item Hypomania Checklist (HCL‐32). The score of MINI (hypo‐) manic episode with mixed features—DSM‐5 Module—Chinese version(MINI‐M‐C) ≥ 2 was considered as the gold standard of mixed features, and the receiver operating characteristic (ROC) curve analysis was used to calculate the optimal cut‐off values of CUDOS‐C score.

**Results:**

The Cronbach's alpha value of CUDOS‐C was 0.898, and the ICC of CUDOS‐C test‐retest was 0.880 (95% CI: 0.812‐0.923, *p *< .05).The CUDOS‐C score was significantly correlated with PHQ‐9 score (*r* = 0.893, *p* = .000), but not with HCL‐32 score(*r* = 0.088, *p *= .364).The area under ROC curve was 0.909 (95% CI: 0.855 to 0.963, *p *< .001) for CUDOS‐C identifying mixed features in mania. The optimal cut‐off value was 11 with a sensitivity of 0.854 and a specificity of 0.868. The CUDOS‐C (score ≥ 12) identified 40.4% of the patients with mixed features, which was higher than those diagnosed by clinicians (18.3%) and screened using MINI‐M‐C (37.6%).

**Conclusions:**

The results indicate the CUDOS‐C is a reliable and valid self‐administered questionnaire for assessing depressive symptoms and screening patients with mixed mania.

## Graphical abstract

This study suggested that the area under the receiver operating characteristic (ROC) curve of the Chinese version of the Clinically Useful Depression Outcome Scale (CUDOS‐C) was 0.909 (95% CI: 0.855 to 0.963, P< 0.001) for identifying mixed features in patients with manic episode. The optimal cut‐off value was 11 with a sensitivity of 0.854 and specificity of 0.868. The CUDOS‐C (score ≥ 12) determined 40.4% of the patients with mixed features, whose detection rate was higher than clinicians' diagnoses (18.3%). The results indicate the CUDOS‐C is a reliable and valid self‐administered questionnaire for assessing depressive symptoms and screening patients with mixed mania.

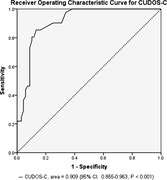

## 1 INTRODUCTION

Mixed state or mixed episode is a kind of mood disorder with a co‐occurrence or overlapping state of (hypo‐) manic and depressive episodes. At the end of the 19th century, Kraepeline first put forward the concept and definition of mixed state. Subsequently, mixed states have been almost completely neglected for decades under the influence of the idea that the combination of manic and depressive features should not be considered a mood disorder (Verdolini et al., 2015).The third and fourth editions of the United States diagnostic and statistical manual of mental disorders (DSM‐III and DSM‐IV) and the tenth edition of the international classification of diseases (ICD‐10) classified mixed states as a subtype of bipolar I disorder. However, the criteria of mixed states in DSM or ICD is too strict to meet in clinical practice, which leads to misdiagnosis and inappropriate treatments, and consequently poor prognostic outcomes (Bipolar Disorder Collaboration Group, Psychiatric Branch of Chinese Medical Association, 2018 ; Jain et al., 2017).

The Systematic Treatment Enhancement Program for Bipolar Disorder (STEP‐BD) investigated the manic symptoms in 1380 patients with bipolar depressed episodes, and found that more than two‐thirds of the subjects had concomitant manic symptoms. Still, only 14.8% of the patients met the DSM‐IV criteria for mixed episodes (Goldberg et al., 2009). Based on the updated clinical practice and research data of diagnosis and treatment for bipolar disorders, the fifth edition of DSM (DSM‐5) has changed the terms “mixed states and mixed episodes” into the “with mixed features” specifier (MFS) of (hypo‐) manic and major depressive episodes, which could capture subthreshold and nonoverlapping symptoms of the opposite poles and form a continuous spectrum diagnosis from a manic episode to a depressive episode (Fagiolini et al., 2015; Perugi et al., 2014; Vieta & Valentí, 2013).

A recent meta‐analysis showed that 35% participants had MFS in those patients diagnosed with bipolar (hypo‐) mania or bipolar depression according to the DSM‐5 diagnostic criteria, and the proportion of patients with MFS in unipolar depression was as high as 24% (Vázquez et al., 2018). However, there are still controversies about the validity and utility of MFS, and the worries about overdiagnosis and overtreatment of bipolar disorders are brought out (Koukopoulos et al., 2013; Verdolini et al., 2014; Vieta & Valentí, 2013). So far, most of the published data were retrospective, or used alternative definitions rather than DSM‐5diagnostic criteria for MFS. More systematic and prospective studies are in pressing needs to fully assess the effects and implications associated with the use of MFS in clinical practice (McIntyre et al., 2013; Perlis et al., 2014; Verdolini et al., 2015).

Due to the diverse and complex clinical manifestations and lack of screening indicators, mixed features are usually insufficiently identified and diagnosed. We recently conducted a multicenter survey and found that the proportion of MFS among (hypo‐) manic patients diagnosed by the clinicians using the DSM‐5 criteria was only 18% in China, which was far lower than the reported data (Fei et al., 2020). It is necessary to adopt screening tools to help clinicians make an acute diagnosis for MFS (Fei et al., 2020). More than two scales are often used to evaluate the different symptoms of depression, mania and other dimensions in patients with mood disorders (especially bipolar disorders), which would cause prolonged scale assessments and noncompliance in patients.

The clinically useful depression outcome scale supplemented with questions for the DSM‐5 MFS (CUDOS‐M) includes three dimensions: depressive symptoms, manic symptoms, and functional impairment (Zimmerman et al., 2014). Recently, one study has explored the reliability and validity of the Chinese version of CUDOS‐M (CUDOS‐M‐C) for the Chinese patients with depressive episodes, and the findings supported that CUDOS‐M‐C could effectively screen those patients with mixed depression (Du et al., 2021). Our study aims to explore the reliability, validity, and feasibility of the depressive dimensionality of CUDOS‐M, that is, the Clinically Useful Depression Outcome Scale (CUDOS) (Zimmerman et al., 2008), to identify the mixed features in manic episodes and then find that CUDOS‐M as a simple screening tool can simultaneously identify both mixed depression and mixed mania.

## 2 MATERIALS AND METHODS

### 2.1 Participants

Convenience sampling method was adopted to recruit outpatients or inpatients from the division of affective disorders (mood disorders) of Hongkou District Mental Health Centre, Shulan (Hangzhou) Hospital, Shenzhen Mental Health Centre, Anhui Mental Health Center, Shanghai Mental Health Center, and Wuxi Mental Health Center from March 2018 to April 2020 (Fei et al., 2020). The inclusion criteria of patients were as follows: (1) aged from 18 to 65 years old; (2) currently diagnosed with (hypo‐) manic episode of bipolar disorder according to the DSM‐5 criteria; (3) with or without treatment history; (4) with an education level of junior high school or higher, and with sufficient cognitive ability to understand informed consent and research content. Patients with (hypo‐) manic episode caused by substances or drugs, physical diseases, or other major psychiatric disorders (such as schizophrenia) were excluded from the survey. Besides, patients with manic episodes too severe to cooperate with the assessment of scales, and other conditions which were not suitable for participation in the study were also excluded.

This study was reviewed and approved by the Ethics Committee of Hongkou District Mental Health Center of Shanghai (approval number: 2018‐B04). Before the implementation of any evaluation, all participants provided written informed consent.

### 2.2 Instruments

The original version of CUDOS was developed by Zimmerman et al. and had good consistency with other self‐rating scales of depressive symptoms (Jeon et al., 2017; Trujols et al., 2013; Zimmerman et al., 2008, 2012). Afterwards, the items of manic symptoms were added to form a self‐rating questionnaire composed of 31 items for screening mixed features (mixed depression) in patients with depressive disorders, in which items 1–16 were depressive symptom dimensions, items 17–29 were manic symptom dimensions, and items 30–31 were functional impairment dimensions (Zimmerman et al., 2014). The subjects chose the number that best describes their condition during the past week (including today): 0 = not at all (0 days), 1 = rarely (1‐2 days), 2 = sometimes (3‐4 days), 3 = often (5‐6), 4 = almost always (every day). The three dimensions were scored separately: the higher the score, the more serious the depressive or manic symptoms are. The Chinese version of CUDOS (CUDOS‐C) has been used for screening depressive symptoms in diabetic patients (Hsu et al., 2014). The CUDOS‐C (the depressive symptoms dimension of CUDOS‐M‐C) in this study was used to assess the depressive symptoms in (hypo‐) manic patients.

The Chinese version of Mini International Neuropsychiatric Interview (MINI) 5.0 was used to rereview the clinical diagnosis and ascertain the (hypo‐) manic episode. The Mini International Neuropsychiatric Interview (Hypo‐) Manic Episode with Mixed Features‐ DSM‐5 Module (MINI‐M) is a supplementary module of MINI and a self‐evaluation tool for screening the mixed features in (hypo‐) manic episode (Hergueta & Weiller, 2013). The MINI‐M questionnaire consists of six items and nine 00questions, and is consistent with the definition and diagnostic criteria of DSM‐5 for MFS. Our recent study demonstrated that the Chinese version of MINI‐M(MINI‐M‐C) was reliable and valid for screening mixed features with 2 points as the best cut‐off value in patients with (hypo‐) manic episode, and helpful for raising clinical diagnostic sensitivity of mixed features (Fei et al., 2020).

The Patient Health Questionnaire‐9 (PHQ‐9) and32‐item Hypomania Checklist (HCL‐32) were self‐rated questionnaires to assess depressive symptoms and (hypo‐) manic symptoms, respectively. The two questionnaires have been proved to have a good reliability and validity in their Chinese‐translated version (Wang et al., 2014; Yang et al., 2011) and were used to test the convergent and discriminant validity of CUDOS‐C.

### 2.3 The formulation of CUDOS‐C

Authorization to translate the CUDOS and CUDOS‐M into Chinese was granted by the original author, Professor Zimmerman. Two experienced psychiatrists (one attending physician and one chief physician) with excellent proficiency in English translated the original document and did preliminary proofreading and editing. Then, the experts who are fluent in both English and Chinese reviewed the translation and provided suggestions for revision. After discussion and consensus, the CUDOS‐M‐C (containing CUDOS‐C) was formed. Later, two professionals (one Master of Clinical Psychology and one Deputy Chief Psychiatrist studying in Canada) who had never seen this scale before translated the Chinese version back into English (the language of the original version) and compared the back translated version with the original for further revisions. The back‐translated version was reviewed and proofread by Professor Zimmerman to confirm that the semantic meaning of the translated manuscript is consistent with that of the original. Finally, other members of the research group evaluated the Chinese version and agreed that all items had a clear meaning and were in accordance with the habits of Chinese language expression, thus forming the final version of CUDOS‐M‐C and CUDOS‐C. The CUDOS‐M‐C and CUDOS‐C only got polished with some minor edits for specific words based on Chinese culture and setting to make it more suitable for the Chinese population. The CUDOS‐C includes 16 items without item added or deleted, and each item is scored from 0 (not present) to 4 (severe).

### 2.4 Clinical interview and measurement

The attending physicians in charge of the patients’ diagnosis and treatment used the DSM‐5 diagnostic criteria to determine whether patients had (hypo‐) manic episode and MFS. The participants were interviewed face to face by trained research assistants (including psychiatrists and psychologists). The research assistants used a self‐designed questionnaire to collect demographic information, and the Chinese version of MINI 5.0 to ascertain the (hypo‐) manic episode of the participants. The participants filled out the self‐evaluation questionnaires of CUDOS‐M‐C (CUDOS‐C), MINI‐M‐C, PHQ‐9, and HCL‐32 by themselves. If any items on any questionnaires are missing, the research assistants would withdraw them on the spot from the study. A part of the participants was retested using CUDOS‐C at the end of the first week.

### 2.5 Statistical analysis

All data were analyzed with SPSS version 25.0 statistical analysis software. The categorical data were presented as the number and frequency of observations, and the continuous data were presented as means ± standard deviation (SD) or median (25%, 75% quantile) if without normal distribution. The internal consistency of CUDOS‐C was evaluated using Cronbach's α coefficient and item‐total correlation. The intraclass correlation coefficient (ICC) between the scores at the baseline and at the first weekend was calculated to examine the test‐retest reliability of CUDOS‐C. Spearman correlation coefficient was used to analyze the convergent and discriminant validity by comparing the correlation between CUDOS‐C and PHQ‐9, HCL‐32. Setting the score of MINI‐M‐C ≥ 2as the gold standard, the diagnostic validity of CUDOS‐C for screening mixed features was analyzed using the receiver operating characteristic (ROC) curve, and the sensitivity and specificity were evaluated to obtain the optimal cutoff score. All statistical tests were two‐tailed, and a *p* value < .05 was considered to be statistically significant.

## 3 RESULTS

### 3.1 Demographic and clinical characteristics

A total of 112 questionnaires were distributed to the participants, and 109 valid questionnaires were obtained (response rate as 98.1%). Of the 109 participants included, 60 (55.0%) patients were male and 49 (45.0%) were female, and age was 32.00(23.50, 45.00) years. Ninety‐two (84.4%) patients were diagnosed with manic episode and 17 (15.6%) were diagnosed with hypomanic episode. Of all 109 participants, 80 patients (73.4%) were followed up at the end of the first week.

### 3.2 Internal consistency and test–retest reliability of CUDOS‐C

The Cronbach's alpha value of internal consistency for the CUDOS‐C was 0.898, and the Cronbach's alpha coefficients after deleting each item ranged from 0.877 to 0.906. The corrected item‐total correlations were between0.108 and 0.752 at baseline. The lowest item‐total correlations were for two atypical depressive symptoms [item 4 “increased appetite” (correlation coefficient = 0.108) and item 6 “hypersomnia” (correlation coefficient = 0.425)]. The ICC of CUDOS‐C test‐retest after 1 week in 80 patients was 0.880 (95% CI: 0.812‐0.923,*p *< .05), and the ICC of each item were between 0.393 and 0.883 (*p *< .05). The results of reliability analysis are shown in Table 1.

**TABLE 1:** Internal consistency, corrected item‐total correlation, and test‐retest reliability of CUDOS‐C items

CUDOS‐C item	Cronbach's alpha if item deleted	Corrected item‐total correlation	Test‐retest reliability (n = 80)
1. I felt sad or depressed	0.886	0.530	0.696
2. I was not as interested in my usual activities	0.879	0.715	0.724
3. My appetite was poor and I didn't feel like eating	0.885	0.552	0.532
4. My appetite was much greater than usual	0.906	0.108	0.470
5. I had difficulty sleeping	0.881	0.642	0.724
6. I was sleeping too much	0.891	0.425	0.393
7. I felt very fidgety, making it difficult to sit still	0.882	0.633	0.777
8. I felt physically slowed down, like my body was stuck in mud	0.883	0.663	0.721
9. My energy level was low	0.883	0.606	0.740
10. I felt guilty	0.886	0.522	0.423
11. I thought I was a failure	0.884	0.583	0.659
12. I had problems concentrating	0.877	0.752	0.820
13.I had more difficulties making decisions than usual	0.883	0.611	0.883
14. I wished I was dead	0.885	0.571	0.688
15. I thought about killing myself	0.886	0.549	0.752
16. I thought that the future looked hopeless	0.885	0.552	0.431

### 3.3 Validity analysis of CUDOS‐C

The scores for CUDOS‐C, PHQ‐9, and HCL‐32 scales at baseline were 9.00(3.00, 19.50), 3.00(0, 10.00), and 14.00(8.00, 18.00), respectively. The spearman correlation analysis showed that CUDOS‐C score was significantly correlated with PHQ‐9 score at baseline(*r* = 0.893, *p* = .000), but not with HCL‐32 score at baseline (*r* = .088, *p *= .364).

Of the 109 patients, 18.3% (20/109) patients were diagnosed with MFS in manic or hypomanic episode by clinicians according to the DSM‐5 criteria, and 37.6% (41/109) patients were identified as MFS using MINI‐M‐C (score ≥ 2).

**FIGURE 1:**
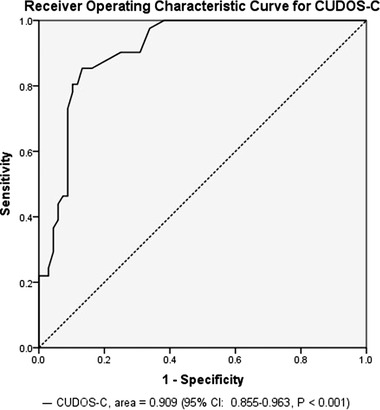
Receiver operating characteristic curve of CUDOS‐C in mixed mania

Setting MINI‐M‐C score ≥ 2 as the gold standard of screening MFS (Fei et al., 2020), the area under ROC curve was 0.909 (95% CI: 0.855 to 0.963, *p *< .001) for CUDOS‐C identifying MFS in patients with (hypo‐) manic episode (Figure 1), which indicated an excellent screening validity. The results of sensitivity and specificity analysis for the CUDOS‐C are shown in Table 2, and the optimal cut‐off value was 11 points by calculating the largest Yoden index. The sensitivity of this value was 0.854 and the specificity was 0.868. Thus, CUDOS‐C score ≥ 12 detected 40.4% (44/109) (hypo‐) manic patients with mixed features.

**TABLE 2:** The sensitivity and specificity of CUDOS‐C for identifying mixed features in mania

CUDOS‐C cut‐off score	Sensitivity	Specificity	Youden index
8/9	0.902	0.721	0.623
9/10	0.902	0.750	0.652
10/11	0.854	0.838	0.692
11/12	0.854	0.868	0.721
12/13	0.805	0.882	0.687
13/14	0.805	0.897	0.702
14/15	0.780	0.897	0.678

## 4 DISCUSSION

Increasing evidences have indicated that mixed features are common in patients diagnosed with bipolar disorder, and are associated with poor outcomes and treatment response (Bipolar Disorder Collaboration Group, Psychiatric Branch of Chinese Medical Association, 2018 ; Jain et al., 2017; Vázquez et al., 2018). Therefore, the development of appropriate standardized assessments for identifying and evaluating mixed features is warranted. This real‐world study explored the psychometric characteristics of the CUDOS‐C in patients diagnosed with (hypo‐) manic episode. The findings revealed that the CUDOS‐C is a valid instrument for assessing symptoms of depression and screening mixed features in patients with manic episode (mixed mania) due to its excellent internal consistency, test‐retest reliability, and screening validity.

A scale with good reliability should satisfy the Cronbach's alpha coefficient of the total scale > 0.80 and that of each dimension >0.60 (Terwee et al., 2007). The overall Cronbach's alpha coefficient and the Cronbach's alpha coefficients after deleting each item for the CUDOS‐C were about 0.90 suggesting an excellent internal consistency, which was similar to that of the original CUDOS, Spanish and Korean versions (Jeon et al., 2017; Trujols et al., 2013; Zimmerman et al., 2008). Though the minor depression symptoms at baseline and the therapeutic effect over 1 week could affect the correlation, the high ICC values (0.39‐0.88) of total scale and each item demonstrated an acceptable test‐retest reliability of the CUDOS‐C. The internal consistency and test‐retest reliability of the CUDOS‐C was also comparable with that of the CUDOS‐C‐M (Du et al., 2021), and superior to the MINI‐M‐C in Chinese patients with mood disorders (Fei et al., 2020).

The Hamilton Rating Scale for Depression (HAMD) and Montgomery and Asberg Depression Rating Scale (MADRS) are the most frequently used scales evaluating depressive symptoms. However, they are clinician administered, requiring training and more time to administer reliably and validly, and only measure decrease in sleep and appetite (Furukawa, 2010; Huijbrechts et al., 1999). The CUDOS consists of 16 items for assessing the depressive symptoms, and measures increase and decrease in sleep or appetite using separate items, which is similar to Quick Inventory of Depressive Symptomatology (Rush et al., 2003; Zimmerman et al., 2012), and different with PHQ‐9 using a single item (Levisz et al., 2019). The atypical depression symptoms (increase in sleep and appetite) had the lowest item‐scale correlations consistent with the results of Korean validation study, that supported increase and decrease in sleep or appetite needs to be evaluated separately in clinical practice (Jeon et al., 2017).

The ROC curve demonstrated that CUDOS‐C acted as a good screening tool for mixed mania at the optimal value as 11. The cut‐off score was lower than those scores (optimal cut‐off values as 19) for identifying depression in patients with type 2 diabetes mellitus and identifying remission in patients with depression (Zimmerman et al., 2012, 2004). The area under ROC curve (0.91), sensitivity (0.85), and specificity (0.87) of CUDOS‐C in these participants were consistent with those of the original CUDOS in patients with major depressive episode (ROC area 0.95, sensitivity 0.88, and specificity 0.88) (Zimmerman et al., 2004), and higher than those in patients with type 2 diabetes mellitus (ROC area 0.84, sensitivity 0.78, and specificity 0.76) (Zimmerman et al., 2012). The discrepancy could be attributed to the disease characteristics of the enrolled participants (Zimmerman et al., 2012). Moreover, the characteristics of CUDOS‐C discriminating mixed features in (hypo‐) manic patients was better than those of MINI‐M‐C (ROC area 0.77, sensitivity 0.80, and specificity 0.71) (Fei et al., 2020).

The available data suggested that depressive symptoms co‐occurred alongside mania in 10−30% of patients (Yatham et al., 2018). The 40% of patients with (hypo‐) manic episode were screened out MFS using CUDOS‐C (score ≥ 12) in this study, which was comparable with the previous findings (35−38%) and higher than the rate of 18% diagnosed by the clinicians using routine psychiatric interview (Fei et al., 2020; Vázquez et al., 2018). While Hergueta and Weiller (2013) reported that 46.5% manic patients had MFS diagnosed by psychiatrists using DSM‐5criteria, and the proportion of MFS increased to 58.6% using MINI‐M questionnaire. The reasons for this discrepancies may be that the latter study adopted stricter standards for enrolled patients (e.g., bipolar I disorder and manic episode) and clinical experience of clinicians (qualified for 3 to 30 years and treated a minimum of 15 bipolar I disorder patients per month) (Hergueta & Weiller, 2013). Overall, these screening tools are helpful for raising the clinical recognition of mixed features in depression and bipolar disorder.

The strength of this study was that we used a real‐world and multiple‐center design to explore the multidimensional reliability and validity of CUDOS‐C. However, several limitations of the present study need to be considered when interpreting the findings. First, the sample size was too limited to conduct more inferential statistical tests to compare the results between different demographic and clinical characteristics, for example, hypomania and mania, outpatients and inpatients, acute episode and partial remission phase. Second, we examined the correlation between CUDOS‐C and self‐report measures (PHQ‐9 and HCL‐32), but we did not examine the association with a clinician measure of symptomatology such as HAMD, MADRS and Young Mania Rating Scale. Finally, bipolar disorder is well known to have a high rate of comorbidity. Any co‐occurring psychiatric disorders were not listed as exclusion criteria, and comorbid diagnoses/symptoms were not assessed, which could influence the sample representativeness and findings generalization.

## 5 CONCLUSIONS

Taken together, the results of this study indicate that CUDOS‐C is a reliable and valid self‐administered questionnaire for assessing depressive symptoms and screening mixed features in bipolar patients with hypomania or mania. If integrated with the manic symptom dimensions of CUDOS‐M‐C, it is useful for effectively measuring concurrent mixed mania and mixed depression. It can easily be incorporated into routine psychiatric evaluations for patients with bipolar disorder and will help clinicians to identify bipolar disorder with mixed features, and prescribe more appropriate treatment for these patients, for example, antidepressants need to be avoided. While the results of this validation study are encouraging, they require replication in large samples with different demographic and clinical characteristics.

## DISCLOSURES

The authors declare no conflict of interest.
